# Trends in the prevalence and management of diabetes in Korea: 2007-2017

**DOI:** 10.4178/epih.e2019029

**Published:** 2019-07-04

**Authors:** Ji-Yeon Shin

**Affiliations:** Department of Preventive Medicine, School of Medicine, Kyungpook National University, Daegu, Korea

**Keywords:** Diabetes mellitus, Prevalence, Awareness, Glycated hemoglobin A, Blood pressure, Cholesterol

## Abstract

**OBJECTIVES:**

This study analyzed Korea National Health and Nutrition Examination Survey data from 2007 to 2017 to assess trends in the prevalence, treatment, and control of diabetes in Korean adults ≥30 years of age.

**METHODS:**

Prevalent diabetes was defined as a fasting plasma glucose level ≥126 mg/dL, self-reported use of anti-diabetic treatment (insulin or oral anti-diabetic drugs), or diabetes diagnosis by a physician. Target levels were defined as glycosylated hemoglobin <6.5% or <7.0%, blood pressure <130/80 mmHg, and total cholesterol <200 mg/dL. All survey waves were age-standardized to the 2005 Korean census population.

**RESULTS:**

Diabetes prevalence increased from 9.6% in 2007-2009 to 10.8% in 2016-2017 (p<0.001). Impaired fasting glucose prevalence significantly increased in both genders and almost every age group. Diabetes awareness and glycemic control did not show an increasing trend; however, the treatment rate and proportion of people diagnosed with diabetes achieving target blood pressure and total cholesterol levels improved from 57.2% to 63.5% (p=0.008), from 41.1% to 53.2% (p<0.001), and from 65.0% to 78.0% (p<0.001), respectively.

**CONCLUSIONS:**

From 2007 to 2017, the prevalence of diabetes increased moderately in Korea, whereas the diabetes treatment rate and the proportion of people diagnosed with diabetes achieving target blood pressure and total cholesterol levels improved. However, awareness of diabetes and glycemic control require significant improvements. A national-level action plan is required to raise awareness about diabetes and prediabetes, with the goal of improving glycemic control and minimizing the occurrence of adverse health outcomes.

## INTRODUCTION

Diabetes is a significant global public health issue and its burden is projected to increase [1]. In South Korea (hereafter Korea), diabetes is a major contributor to the burden of disease [2,3], affecting approximately 5 million Korean adults in 2016 [4]. From the 1960s to the late 1990s, the prevalence of diabetes in Korea rapidly increased from less than 1% to 6-9% [5]. From 1998 to 2005, the prevalence of diabetes in adults aged ≥30 years stabilized at 9-11% based on data from the Korea National Health and Nutrition Examination Survey (KNHANES) [6].

The Health Plan 2020 (HP2020), the Korean national vision for health promotion, aimed to maintain the diabetes prevalence at 11.0% and to improve the awareness, treatment, and control of diabetes to 85%, 65%, and 35%, respectively, among adults with diabetes by 2020 [7]. However, trends in the diabetes rates in the last decade have not been analyzed. Studies on trends in the awareness, treatment, and control of diabetes are even rarer.

This study sought to examine whether trends of diabetes prevalence and management indices in the last 10 years are approaching the targets of HP2020. To update the information on trends in diabetes prevalence in Korea, most recently reported for 1998-2005 [6], this study aimed to investigate changes in diabetes prevalence, awareness, treatment, and control among Korean adults aged ≥30 years using KNHANES data between 2007 and 2017. We further assessed diabetes trends according to subpopulation by examining age-specific, gender-specific, income level-specific, and body mass index (BMI)-specific prevalence rates of diabetes. We also aimed to investigate changes in the prevalence of impaired fasting glucose (IFG) among Korean adults, and changes in control of blood pressure (BP) and cholesterol levels among patients with diabetes to examine the likelihood of achieving the HP2020 goals. This analysis of the diabetes management status at a national level could provide valuable insights into the future direction of diabetes management in Korea.

## MATERIALS AND METHODS

### Study population

The KNHANES is a series of nationally representative surveys of the non-institutionalized Korean population conducted by the Korea Centers for Disease Control and Prevention (KCDC). The survey volunteers were selected using a stratified multistage probability sampling design. It started in 1998, and starting in 2007, the survey became a year-round investigation employing a rolling sample design. Because a rolling sample design is used, the annual data within each wave can be integrated if a certain sample is insufficient to produce stable statistics for analyzing specific groups. We used data from the fourth (2007-2009), fifth (2010-2012), sixth (2013-2015), and the seventh (2016-2017) KNHANES (the data from 2017 were the most recent). Details of the KNHANES have been described elsewhere [8]. The KNHANES was approved by the KCDC Institutional Review Board, and all subjects provided written informed consent.

In this study, we included all adults aged ≥30 years who had undergone phlebotomy after a minimum 8-hour fasting period (n=13,931 [5,931 men; 8,018 women], 14,665 [6,272 men; 8,383 women], 12,289 [5,253 men; 7,036 women], and 10,131 [4,454 men; 5,677 women] in the 2007-2009, 2010-2012, 2013-2015, and 2016-2017 KNHANES, respectively). A standardized questionnaire was used to collect information on participants’ socio-demographic characteristics and medical history. For the income level, the equivalent household monthly income, calculated as the monthly household income divided by the square root of the number of persons in the household, was categorized into quartiles (low, mid-low, mid-high, and high) by year and gender. Weight and height were measured using standardized protocols, and BMI was calculated as the weight in kilograms divided by the height in meters squared. BMI was further categorized into <25.0 kg/m^2^, 25.0-29.9 kg/m^2^, and ≥30.0 kg/m^2^. The details of the laboratory analytic methods and quality control have been described elsewhere [9,10].

### Definitions

Prevalent diabetes was defined as a fasting plasma glucose level of ≥126 mg/dL, current anti-diabetic treatment (either insulin or oral anti-diabetic drugs), or a previous diagnosis of diabetes made by a physician. IFG was defined as a fasting plasma glucose level ≥100 mg/dL and less than 126 mg/dL. Even if the IFG criteria were met, we excluded prevalent diabetes cases (as defined above) from the prevalent IFG cases.

Diabetes awareness was defined as subjects who had been diagnosed with diabetes by a physician among those with prevalent diabetes. This definition is the same as “known cases of diabetes” in the study of Choi et al. [6].

Treatment of diabetes was defined as the subjects using a pharmacological treatment (either insulin, oral anti-diabetic drugs, or both) for diabetes among those with prevalent diabetes.

We used two definitions for the control of diabetes, as follows: (1) the proportion of patients with a glycosylated hemoglobin (A1C) level <6.5% among those diagnosed with diabetes by a physician (known cases of diabetes), as suggested by the International Diabetes Federation [11], and (2) the proportion of patients with an A1C level <7.0% among those diagnosed with diabetes by a physician (known cases of diabetes), according to the American Diabetes Association standard [12].

Control of hypertension was defined as a systolic BP<130 mmHg and a diastolic BP <80 mmHg among those diagnosed with diabetes by a physician, according to the American Diabetes Association standards of medical care [13,14]. Control of total cholesterol was defined as a total serum cholesterol level <200 mg/dL among those diagnosed with diabetes by a physician, according to the National Cholesterol Education Program Adult Treatment Panel III target level [14,15].

### Data analysis

According to the KCDC guideline [10], all analyses were performed using appropriate sampling weights to obtain accurate estimates representative of the non-institutionalized Korean population. Data were analyzed using SAS version 9.4 (SAS Institute Inc., Cary, NC, USA). We analyzed the prevalence, awareness, treatment, and control of diabetes, and the achievement of BP and total cholesterol target levels, further stratified by gender, age, income level, and BMI category. For all categories, we calculated weighted percentages and standard errors.

All stratified estimates, except the age-specific prevalence, were age-standardized to allow comparison across the different survey waves. For diabetes and IFG, the prevalence estimates were age-standardized to the 2005 Korean census population, using the following age groups and weights (30-39 years, weight 0.305422673; 40-49 years, weight 0.290505987; 50-59 years, weight 0.182221242; 60-69 years, weight 0.128706547; and ≥70 years, weight 0.093143551). For awareness, treatment, and control, the estimates were age-standardized to the subpopulation of persons who had diabetes in the 2005 KNHANES [16]. In 2005, the diabetes prevalence was 1.4% in persons aged 30-39 years, 7.4% in those aged 40-49 years, 14.0% in those aged 50-59 years, 18.1% in those aged 60-69 years, and 17.9% in those aged ≥70 years [17]. The calculated weights were 0.046857872, 0.235580891, 0.279563386, 0.255289214, and 0.182708637, respectively. We repeated the analysis without age standardization, and those results are presented in Supplementary Materials 1-3.

To analyze the trends over time, we used weighted logistic regression by including the midpoint of each survey period as a continuous variable. The statistical significance of the differences in the age-adjusted prevalence between the survey years was determined using the ILINK option in the SAS PROC SURVEYLOGISTIC procedure. We considered a 2-tailed p-value <0.05 as indicative of statistical significance.

### Ethics statement

Data from the KNHANES survey are made publicly available through the KNHANES website (http://knhanes.cdc.go.kr). Thus, ethical approval was not required for this study.

## RESULTS

The age-standardized prevalence of diabetes in adults aged ≥30 years was 9.6% in 2007-2009 and 10.8% in 2016-2017, showing an increasing trend (p<0.001) (Table 1). The crude prevalence was 10.0% in 2007-2009 and 12.7% in 2016-2017 (Supplementary Material 1). When stratified by age, the prevalence of diabetes showed an increasing trend only among people aged ≥70 years (p for trend <0.001) (Table 1). In 2016-2017, about 3 in 10 (28.5%) adults aged ≥70 years had diabetes (Supplementary Material 1). When stratified by income level, diabetes prevalence increased only in the lowest quartile of monthly household income (p for trend<0.001) (Table 1). When stratified by BMI, a significant increasing trend was observed for those with a BMI ≥30 kg/m^2^ (p for trend=0.023) (Table 1).

**Figure 1.**
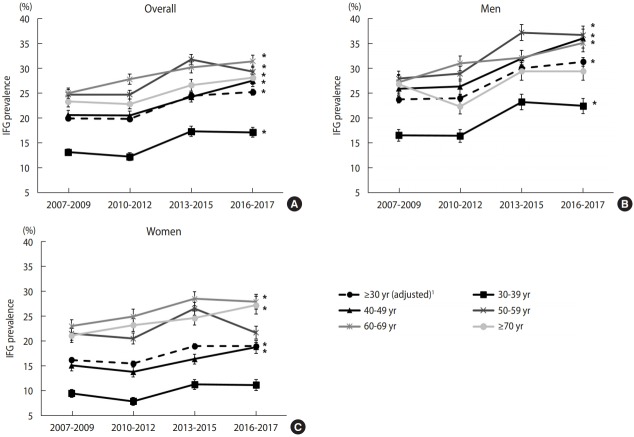
Weighted prevalence of impaired fasting glucose (IFG) among Korean adults aged ≥30 years according to age group and gender, 2007-2017 (A) overall, (B) men, and (C) women. ^1^ Estimates were age-adjusted by direct standardization to the 2005 Korean census population. *p<0.05: p for trend values, which were derived using weighted logistic regression by including the midpoint of each survey period as a continuous variable.

Over the past decade, the prevalence of IFG significantly increased in both genders and almost every age group (Figure 1) (Supplementary Material 2). The unadjusted prevalence of IFG was 20.2% in 2007-2009 and 26.3% in 2016-2017. The IFG prevalence was higher in men than in women, and it has increased more sharply in men (23.7% to 31.3% in men; 16.2% to 19.0% in women; from 2007-2009 to 2016-2017). The IFG prevalence showed a marked increasing trend even among men aged 30-39 years (p for trend<0.001). In 2016-2017, the IFG prevalence in men aged 30-39 was 22.4%, meaning that 1 in 5 men had IFG. Moreover, the IFG prevalence increased by nearly 10 percentage points among men aged 40-59 years over the past decade (men aged 40-49 years: 25.9% in 2007-2009 and 36.0% in 2016-2017; men aged 50-59 years: 27.9% in 2007-2009 and 36.7% in 2016-2017). The IFG prevalence in women aged 30-49 years was only about half that in men, and it did not show a significant increase over time in women aged 30-39 and 50-59.

**Figure 2.**
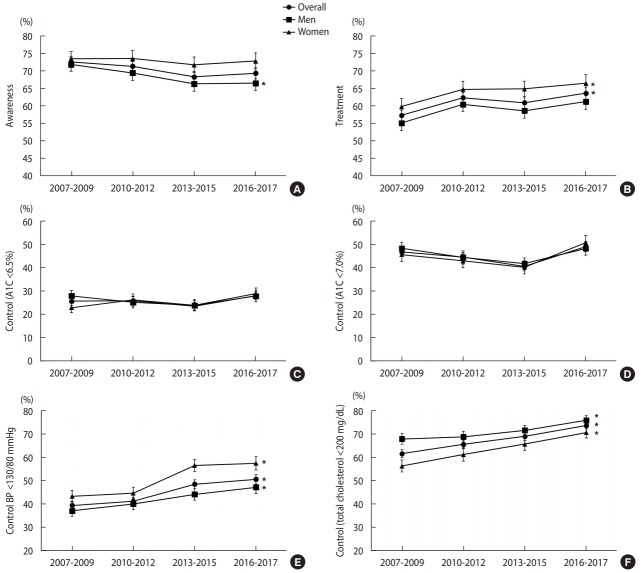
Age-standardized weighted proportion of diabetes awareness, treatment, and control among Korean adults with diabetes aged ≥30 years, 2007-2017 (A) awareness, (B) treatment, (C) control (A1C<6.5%) (D) control (A1C<7.0%), (E) control (BP <130/80 mmHg), and (F) control (total cholesterol <200 mg/dL). All estimates were age-standardized to the subpopulation of persons who had diabetes in the KNHANES 2005. A1C, glycosylated hemoglobin; BP, blood pressure. *p<0.05: p for trend values, which were derived using weighted logistic regression by including the midpoint of each survey period as a continuous variable.

The crude rate of diabetes awareness was 72.3% in 2016-2017 (Supplementary Material 3). In men, diabetes awareness decreased over time (p for trend=0.041) (Figure 2) (Supplementary Material 4). In other words, undiagnosed diabetes increased among men over the past decade. The crude awareness was higher among women than among men by 10 percentage points (Supplementary Material 3).

The proportion of persons receiving anti-diabetic drug treatment (either oral anti-diabetic drugs, insulin, or both) among those with prevalent diabetes increased in the overall population (p for trend=0.008) (Figure 2) (Supplementary Material 4). The crude treatment rate was 62.4% in men and 72.0% in women in 2016-2017 (Supplementary Material 3).

The proportion of persons with an A1C level of <6.5% among those diagnosed with diabetes by physicians was 28.7% in men and 31.4% in women in 2016-2017 (Supplementary Material 3). The proportion of persons with an A1C level of <7.0% was 50.4% in men and 53.4% in women in 2016-2017 (Supplementary Material 3). Although the control rate did not show a significant linear trend over time in both genders (Figure 2) (Supplementary Material 4), the control rate in 2016-2017 for women was significantly higher than that in 2007-2009 or 2013-2015 (between 2007-2009 and 2016-2017, p=0.036, A1C<6.5%; between 2013-2015 and 2016-2017, p=0.004, A1C<7.0%; data not shown).

The age-standardized overall proportion of persons who achieved the target BP significantly increased from 41.1% in 2007-2009 to 53.2% in 2016-2017 (Figure 2) (Supplementary Material 4). The increase was significant in both genders. The age-standardized proportion of persons who achieved the target total cholesterol level also significantly increased in both genders; it was 80.4% in men and 74.8% in women in 2016-2017 among those diagnosed with diabetes by physicians (Figure 2) (Supplementary Material 4). The target BP was more frequently achieved in women, and the target total cholesterol level was more frequently achieved in men.

## DISCUSSION

In this representative sample of non-institutionalized Korean adults, the crude prevalence of diabetes in adults aged ≥30 years increased from 10.0% to 12.7% from 2007 to 2017. The age-standardized prevalence also showed a moderate increasing trend, from 9.6% to 10.8%. Among diabetes awareness, treatment, and control, only the treatment rate showed an increasing trend.

Although the prevalence of diabetes has shown a moderate increase, the prevalence of IFG has shown a significant increase of more than 5 percentage points over the past decade. When stratified by age, the prevalence of diabetes increased only in adults aged ≥70 years. However, the IFG prevalence increased in almost all age groups. Every year, 5-10% of people with prediabetes could progress to diabetes [18]. Moreover, IFG is associated with an increased risk of cardiovascular disease or diabetic microvascular lesions [18,19]. In order to reduce the burden of disease caused by diabetes in the future, it is necessary to systematically manage the prediabetes stage. Fortunately, lifestyle modifications can prevent the progression from prediabetes to diabetes, with evidence of a 40–70% relative-risk reduction [18].

We observed gender-related differences in IFG prevalence, diabetes prevalence, awareness, and management status. The prevalence of diabetes was lower in women than in men, and the proportion of awareness, treatment, adequate glucose control, and BP control was higher in women. The awareness in men has decreased over the past decade. The treatment rates only increased in women over the past decade. As a result, the proportion of individuals with adequate glucose control showed a statistically significant increase only in women (when the control rates of the previous survey years were compared pairwise). Gender-related differences have commonly been observed in studies from many other countries. In China and Kazakhstan, women showed higher levels of diabetes awareness and treatment [20-22]. A USA study likewise reported higher awareness in women [23]. Similar results have been found for hypertension, with men reported to have lower levels of awareness and treatment of hypertension [24,25]. Gender-related differences have been reported for health behaviors and health attitudes [26,27]. In general, women were more likely to be motivated to participate in health-promoting activities and to develop healthy habits [27].

The increase in IFG prevalence in young men can be interpreted in this context. Our results showed that the IFG prevalence was more than twice as high in men in their 30s than in women, and it showed a significant increase only in men (Supplementary Material 2). This is assumed to be closely associated with the health-related lifestyle of men of that age group. According to the 2016 health statistics in Korea [17], the smoking rate, daily smoking rate, and high-risk drinking rate in men were 51.5%, 43.9%, and 23.5%, respectively, in the 30-39 age group, while the corresponding rates in women were 7.6%, 4.9%, and 8.6%, respectively, demonstrating considerable gender differences in health behaviors.

It is notable that a marked increase in diabetes prevalence was only observed in the lowest quartile of income level. This result implies increased socioeconomic inequality in diabetes prevalence. Low socioeconomic status (SES) has recently been recognized as a risk factor for diabetes [28]. Income level was associated with diabetes even after adjustment for known risk factors of diabetes, such as age, gender, level of education, BMI, physical activity, and smoking [29]. It is not yet fully understood how SES increases the risk of diabetes. However, healthcare accessibility, access to healthy food and exercise, occupational opportunities, and personal lifestyle choices are thought to play complex roles in the development of diabetes [28,30]. Further research is needed to examine the factors contributing to the worsening socioeconomic inequalities in the prevalence of diabetes in Korea.

Glycemic control did not show a consistent increasing trend in the 2007-2017 period, although women had higher levels of glycemic control in 2016–2017 than in 2013-2015. It seems that it is difficult to improve or maintain glycemic control at a certain level in the population. In the USA, the glycemic control rate (percentage of A1C level <7.0% among those diagnosed with diabetes) increased from 44.3% in 1999-2002 to 56.8% in 2003-2006. However, it decreased by 4.6% between 2003-2006 and 2007-2010 [31]. In Canada, the glycemic control rate (A1C level <7.0%) of patients with diabetes managed by primary care physicians was 51%. However, the longer the diabetes duration was, the lower the control rates were, despite the increased treatment rate [32]. In Korea, it may become more difficult to increase the proportion of those who achieve target glycemic levels due to the continuing increase in the elderly population, with a longer duration of the disease. In this respect, the recent improvement in the glycemic control rate in women is encouraging.

There was significant improvement in BP and total cholesterol control from 2007 to 2017. It is difficult to make a direct comparison; however, the frequency of BP control (<130/80 mmHg) in 2016-2017 in Korea (53.2%) was higher than that of 48.3% in 2003-2004 [14] or 51.3% in 2007-2010 in the USA [31]. Total cholesterol control was much higher than in the USA (78.0% vs. 50.4%) [14]. In Korea, about 85% of patients with prevalent diabetes are concurrently treated for either high BP or dyslipidemia [14]. The improved BP and cholesterol control among patients with diabetes may be due to better publicity and effective clinical guidelines on the management of diabetes [33]; increased number of people receiving treatment for hypertension, diabetes, and dyslipidemia together [34]; and an increase in statin prescriptions [34].

The prevalence of diabetes and impaired glucose tolerance in adults is projected to increase globally, with the highest increase expected in low-income and middle-income countries [35]. Korea is currently classified as a high-income country, and according to the diabetes prevalence among those aged 20-79 years, it was ranked 135th out of 221 countries in descending order in 2017, when age-standardized with the world population [36]. As is the case for other high-income countries, Korea is also thought to have a moderate increasing trend in the prevalence of diabetes, which peaked among those ≥75 years of age, whereas the prevalence peaked in the 60-74 age groups in middle-income countries and in the 55-64 age groups in low-income countries [37]. Considering the aging trend in Korea, the burden of diseases caused by the increased prevalence of diabetes in the elderly population is expected to rise sharply. To reduce the national burden, it is necessary for the government to actively prepare a national policy to delay the onset of diabetes complications as much as possible in elderly patients. In addition, considering the findings of our study, it is necessary to determine the target groups (such as men or low-SES individuals, who have high prevalence and low management rates) and to provide detailed programs to promote diabetes management. For example, implementing a diabetes management program in the workplace may help vulnerable populations to recognize and manage this disease.

This study had some limitations. First, diagnosed diabetes was determined by self-reporting and was not verified by medical records. Second, most participants only had a single fasting plasma glucose measurement because they participated in the KNHANES examination once, although the American Diabetes Association recommends repeated measurements after a positive result for A1C, fasting plasma glucose, or 2-hour plasma glucose test. Therefore, there may have been instances of misclassification in our study, such that participants who did not have diabetes were categorized as having prevalent diabetes. Third, the denominators of glucose control, BP control, and total cholesterol control all included individuals with diabetes diagnosed by a physician. However, when conducting age standardization, we used the subjects who had prevalent diabetes in the 2005 KNHANES as a standard population, instead of the subjects diagnosed with diabetes by a physician. In 2005, the number of persons diagnosed with diabetes by physicians was 5 among participants in their 30s and 47 among participants in their 40s; thus, it was unreasonable to use this as a standard population because the number of persons per age group was very small and unstable. As a result, the age-standardized glucose, BP, and total cholesterol control levels may have been overestimated or underestimated in the age standardization process. However, using the population with prevalent diabetes as a standard population is sufficient for the purpose of wave-to-wave comparisons with the same age structure during the 2007-2017 periods. Fourth, in the HP2020, the denominator of glucose control was prevalent diabetes cases. However, we used diabetes cases diagnosed by a physician as the denominator of glucose control, because we thought it was more appropriate from a public health perspective to investigate glucose control in persons diagnosed with diabetes who were aware of their status; we wanted to make the proportion of glucose control comparable by using the same definitions as used in previous studies [6,14]. Therefore, compared to the HP2020 findings, this study may have overestimated the results, making caution necessary in comparisons. Nonetheless, the interpretation should not be very problematic, as our results are lower than the HP2020 targets. Fifth, dyslipidemia management standards for patients with diabetes are generally based on low-density lipoprotein (LDL)-cholesterol levels [13]. However, in this study, we examined the control of total cholesterol instead of LDL-cholesterol. In 2009, the KNHANES began measuring LDL-cholesterol using the direct method; however, this test was not performed for all subjects undergoing blood tests. Thus, LDL-cholesterol would have to be calculated using the Friedewald formula, and data on the calculated LDL-cholesterol levels are not available in the KNHANES database. Moreover, the Friedewald formula performs poorly in some situations, such as extreme triglyceride levels (≥400 mg/dL) [38]. Even if the triglyceride level is <400 mg/dL, the use of this formula is not recommended in patients with diabetes [39]. Therefore, we investigated the control of total cholesterol using the criterion of <200 mg/dL, as in the study by Ong et al. [14]. If conditions permit, it may be necessary to investigate LDL-cholesterol control in diabetes patients in future studies.

Despite all these limitations, this study offers meaningful insights into long-term trends in the burden and control of diabetes using rigorously collected national population-based data. The findings could provide useful insights into future healthcare planning and the design of appropriate strategies, both in Korea as well as in countries with similar demographic and health system structures.

In conclusion, from 2007 to 2017, the prevalence of diabetes increased moderately in Korea, whereas the diabetes treatment rate and the proportion of people diagnosed with diabetes who achieved target BP and total cholesterol levels improved. However, the prevalence of impaired fasting glucose increased significantly in nearly every age group. Awareness of diabetes and the level of glycemic control all require significant improvements. The goal of HP2020 of maintaining the prevalence of diabetes at 11.0% by 2020 is likely achievable, as is the treatment rate target of 65%. However, the 85% and 35% targets for awareness and glycemic control are unlikely to be achieved by 2020, considering the 69.2% and 28.0% rates in 2016-2017, respectively. A national-level integrated action plan is required to raise awareness about diabetes and prediabetes with the goal of improving glycemic control and minimizing the occurrence of adverse health outcomes.

## Figures and Tables

**Table 1.** Age-standardized weighted diabetes prevalence among Korean adults aged ≥30 years, 2007-2017

Variables	2007-2009	2010-2012	2013-2015	2016-2017	p for trend^1^
No. with diabetes^2^	1,536	1,700	1,590	1,469	-
Overall					
Adjusted	9.6 (0.3)	9.4 (0.3)	10.2 (0.3)	10.8 (0.4)	<0.001
Gender					
Men	10.9 (0.4)	11.0 (0.4)	12.1 (0.5)	12.6 (0.5)	0.003
Women	8.2 (0.3)	7.9 (0.3)	8.3 (0.4)	9.0 (0.4)	0.023
Age-group (yr)^3^					
30-39	2.7 (0.3)	2.4 (0.3)	2.5 (0.4)	2.4 (0.5)	0.637
40-49	6.5 (0.5)	6.1 (0.6)	7.2 (0.6)	7.3 (0.7)	0.170
50-59	12.6 (0.7)	13.2 (0.7)	11.4 (0.7)	14.7 (0.9)	0.158
60-69	21.2 (0.9)	19.2 (0.8)	22.4 (1.0)	20.7 (1.1)	0.777
≥70	19.9 (1.0)	22.3 (1.0)	25.2 (1.2)	28.5 (1.3)	<0.001
Income level^4^					
Low	11.4 (0.8)	12.8 (1.1)	14.0 (1.2)	16.9 (1.3)	<0.001
Mid-low	10.3 (0.6)	9.3 (0.5)	11.5 (0.6)	10.6 (0.7)	0.377
Mid-high	8.9 (0.6)	9. 3(0.6)	9.1 (0.6)	10.3 (0.6)	0.315
High	8.6 (0.6)	9.0 (0.6)	9.5 (0.6)	8.9 (0.6)	0.177
Body mass index (kg/m^2^)^5^					
<25.0	7.4 (0.3)	7.5 (0.3)	8.1 (0.3)	7.6(0.3)	0.059
25.0-29.9	13.0 (0.6)	12.0 (0.6)	12.4 (0.6)	14.5 (0.7)	0.046
≥30.0	21.0 (2.0)	20.3 (1.8)	24.0 (1.9)	26.1 (2.0)	0.023

Values are presented as % (standard error).

1 Derived using weighted logistic regression by including the midpoint of each survey period as a continuous variable.

2 Unweighted total number of cases of diabetes.

3 Age-specific crude rates are presented.

4 Calculated as monthly household income divided by the square root of the number of persons in the household, categorized into quartiles by year and gender.

5 Calculated as the weight in kilograms divided by the height in meters squared.

## References

[1] Shaw JE, Sicree RA, Zimmet PZ (2010). Global estimates of the prevalence of diabetes for 2010 and 2030. Diabetes Res Clin Pract.

[2] Oh IH, Yoon SJ, Seo HY, Kim EJ, Kim YA (2011). The economic burden of musculoskeletal disease in Korea: a cross sectional study. BMC Musculoskelet Disord.

[3] Bloom DE, Chen S, Kuhn M, McGovern ME, Oxley L, Prettner K (2018). The economic burden of chronic diseases: estimates and projections for China, Japan, and South Korea. J Econ Ageing.

[4] Korean Diabetes Association (2018). Diabetes fact sheet in Korea. http://www.diabetes.or.kr/bbs/index.html?sub_menu=&code=e_resource&category=1&gubun=&page=1&number=381&mode=view&order=&sort=&keyfield=&key=.

[5] Cho NH (2014). Diabetes burden and prevention in Korea and the Western Pacific Region. Diabetes Res Clin Pract.

[6] Choi YJ, Kim HC, Kim HM, Park SW, Kim J, Kim DJ (2009). Prevalence and management of diabetes in Korean adults: Korea National Health and Nutrition Examination Surveys 1998-2005. Diabetes Care.

[7] Ministry of Health and Welfare Korea Health Promotion Institute. National Health Plan 2020 in Korea. https://www.khealth.or.kr/healthplan.

[8] Kweon S, Kim Y, Jang MJ, Kim Y, Kim K, Choi S (2014). Data resource profile: the Korea National Health and Nutrition Examination Survey (KNHANES). Int J Epidemiol.

[9] Korea Centers for Disease Control and Prevention (2014). Quality control of the clinical laboratory for Korea National Health and Nutrition Examination Survey 2014, 6th second year.

[10] Korea Centers for Disease Control and Prevention (2019). The guideline for the usage of Korean National Health and Nutrition Survey raw data.

[11] International Diabetes Federation Guideline Development Group (2014). Global guideline for type 2 diabetes. Diabetes Res Clin Pract.

[12] Chamberlain JJ, Rhinehart AS, Shaefer CF, Neuman A (2016). Diagnosis and management of diabetes: synopsis of the 2016 American Diabetes Association Standards of Medical Care in Diabetes. Ann Intern Med.

[13] American Diabetes Association (2012). Standards of medical care in diabetes--2012. Diabetes Care.

[14] Ong KL, Cheung BM, Wong LY, Wat NM, Tan KC, Lam KS (2008). Prevalence, treatment, and control of diagnosed diabetes in the U.S. National Health and Nutrition Examination Survey 1999-2004. Ann Epidemiol.

[15] Expert Panel on Detection, Evaluation, and Treatment of High Blood Cholesterol in Adults (2001). Executive summary of the third report of the National Cholesterol Education Program (NCEP) expert panel on detection, evaluation, and treatment of high blood cholesterol in adults (Adult Treatment Panel III). JAMA.

[16] Crim MT, Yoon SS, Ortiz E, Wall HK, Schober S, Gillespie C (2012). National surveillance definitions for hypertension prevalence and control among adults. Circ Cardiovasc Qual Outcomes.

[17] Korea Centers for Disease Control and Prevention (2017). Korea health statistics 2016: Korea National Health and Nutrition Examination Survey (KNHANES VII-1).

[18] Tabák AG, Herder C, Rathmann W, Brunner EJ, Kivimäki M (2012). Prediabetes: a high-risk state for diabetes development. Lancet.

[19] Kim HK, Kim CH, Kim EH, Bae SJ, Choe J, Park JY (2013). Impaired fasting glucose and risk of cardiovascular disease in Korean men and women: the Korean Heart Study. Diabetes Care.

[20] Yue J, Mao X, Xu K, Lü L, Liu S, Chen F (2016). Prevalence, awareness, treatment and control of diabetes mellitus in a Chinese population. PLoS One.

[21] Li J, Ni J, Wu Y, Zhang H, Liu J, Tu J (2019). Sex differences in the prevalence, awareness, treatment, and control of diabetes mellitus among adults aged 45 years and older in rural areas of northern China: a cross-sectional, population-based study. Front Endocrinol (Lausanne).

[22] Supiyev A, Kossumov A, Kassenova A, Nurgozhin T, Zhumadilov Z, Peasey A (2016). Diabetes prevalence, awareness and treatment and their correlates in older persons in urban and rural population in the Astana region, Kazakhstan. Diabetes Res Clin Pract.

[23] McDonald M, Hertz RP, Unger AN, Lustik MB (2009). Prevalence, awareness, and management of hypertension, dyslipidemia, and diabetes among United States adults aged 65 and older. J Gerontol A Biol Sci Med Sci.

[24] Egan BM, Zhao Y, Axon RN (2010). US trends in prevalence, awareness, treatment, and control of hypertension, 1988-2008. JAMA.

[25] Lee HS, Park YM, Kwon HS, Lee JH, Park YJ, Lim SY (2010). Prevalence, awareness, treatment, and control of hypertension among people over 40 years old in a rural area of South Korea: the Chungju Metabolic Disease Cohort (CMC) Study. Clin Exp Hypertens.

[26] Varì R, Scazzocchio B, D’Amore A, Giovannini C, Gessani S, Masella R (2016). Gender-related differences in lifestyle may affect health status. Ann Ist Super Sanita.

[27] von Bothmer MI, Fridlund B (2005). Gender differences in health habits and in motivation for a healthy lifestyle among Swedish university students. Nurs Health Sci.

[28] Agardh E, Allebeck P, Hallqvist J, Moradi T, Sidorchuk A (2011). Type 2 diabetes incidence and socio-economic position: a systematic review and meta-analysis. Int J Epidemiol.

[29] Hwang J, Shon C (2014). Relationship between socioeconomic status and type 2 diabetes: results from Korea National Health and Nutrition Examination Survey (KNHANES) 2010-2012. BMJ Open.

[30] Blas E, Kurup AS (2010). Equity, social determinants and public health programmes. https://apps.who.int/iris/handle/10665/44289.

[31] Ali MK, Bullard KM, Saaddine JB, Cowie CC, Imperatore G, Gregg EW (2013). Achievement of goals in U.S. diabetes care, 1999-2010. N Engl J Med.

[32] Harris SB, Ekoé JM, Zdanowicz Y, Webster-Bogaert S (2005). Glycemic control and morbidity in the Canadian primary care setting (results of the diabetes in Canada evaluation study). Diabetes Res Clin Pract.

[33] Jeon JY, Kim DJ, Ko SH, Kwon HS, Lim S, Choi SH (2014). Current status of glycemic control of patients with diabetes in Korea: the fifth Korea National Health and Nutrition Examination Survey. Diabetes Metab J.

[34] Korean Society of Lipid and Atherosclerosis (2018). Dyslipidemia fact sheets in Korea, 2018.

[35] Ogurtsova K, da Rocha Fernandes JD, Huang Y, Linnenkamp U, Guariguata L, Cho NH (2017). IDF diabetes atlas: global estimates for the prevalence of diabetes for 2015 and 2040. Diabetes Res Clin Pract.

[36] International Diabetes Federation IDF diabetes atlas - 8th edition [cited 2019May 10]. https://diabetesatlas.org/across-the-globe.html.

[37] Cho NH, Shaw JE, Karuranga S, Huang Y, da Rocha Fernandes JD, Ohlrogge AW (2018). IDF diabetes atlas: global estimates of diabetes prevalence for 2017 and projections for 2045. Diabetes Res Clin Pract.

[38] McNamara JR, Cohn JS, Wilson PW, Schaefer EJ (1990). Calculated values for low-density lipoprotein cholesterol in the assessment of lipid abnormalities and coronary disease risk. Clin Chem.

[39] Rubiés-Prat J, Reverter JL, Sentí M, Pedro-Botet J, Salinas I, Lucas A (1993). Calculated low-density lipoprotein cholesterol should not be used for management of lipoprotein abnormalities in patients with diabetes mellitus. Diabetes Care.

