# Supplementary material for: Trends in the prevalence and management of diabetes in Korea: 2007-2017
Source: Epidemiol Health. 2019 Jul 4;41:e2019029. doi: 10.4178/epih.e2019029 (PMC6702122; doi:10.4178/epih.e2019029)
Supplement: Supplementary file 1 [file epih-41-e2019029-supplementary.pdf]

SUPPLEMENTARY MATERIALS

Supplementary Material 1. Crude weighted diabetes prevalence among Korean adults aged ≥30 years, 2007-2017

	2007-2009			2010-2012			2013-2015			2016-2017		
	n	%	(SE)	n	%	(SE)	n	%	(SE)	n	%	(SE)
Overall												
Unadjusted	2723116	10.0	(0.3)	2845113	10.2	(0.3)	3024909	11.2	(0.3)	4081084	12.7	(0.4)
Sex												
Men	1479051	11.0	(0.5)	1583843	11.5	(0.5)	1718103	13.0	(0.5)	2255053	14.1	(0.6)
Women	1244065	9.0	(0.4)	1261271	8.8	(0.4)	1306806	9.5	(0.4)	1826031	11.3	(0.5)
Age-group (years)												
30-39	205306	2.7	(0.3)	174982	2.4	(0.3)	163647	2.5	(0.4)	170050	2.4	(0.5)
40-49	505308	6.5	(0.5)	467584	6.1	(0.6)	512126	7.2	(0.6)	603290	7.3	(0.7)
50-59	714265	12.6	(0.7)	851290	13.2	(0.7)	766879	11.4	(0.7)	1150836	14.7	(0.9)
60-69	750880	21.2	(0.9)	708461	19.2	(0.8)	873188	22.4	(1.0)	1016621	20.7	(1.1)
≥70	547357	19.9	(1.0)	642796	22.3	(1.0)	709070	25.2	(1.2)	1140287	28.5	(1.3)
Income level [1]												
Low	774897	17.3	(0.8)	822345	18.1	(0.9)	838513	20.7	(1.0)	1290084	25.6	(1.1)
Mid-low	721684	11.1	(0.7)	741766	9.7	(0.5)	857328	13.0	(0.7)	937200	12.3	(0.7)
Mid-high	561763	7.3	(0.5)	662164	8.4	(0.6)	653248	8.1	(0.6)	918150	9.8	(0.7)
High	558501	7.0	(0.5)	582904	7.6	(0.5)	659020	8.0	(0.5)	898070	9.0	(0.6)
BMI (kg/m^2) [2]												
<25.0	1323405	7.4	(0.3)	1481690	8.1	(0.3)	1562335	8.8	(0.4)	1867206	9.2	(0.4)
25.0-29.9	1171833	14.2	(0.6)	1132805	13.3	(0.6)	1161579	14.4	(0.7)	1755254	17.5	(0.8)
≥30.0	207210	20.4	(2.2)	221253	19.4	(1.9)	299315	23.5	(2.0)	442745	26.3	(2.1)

SE, standard error; BMI, body mass index,

[1] Calculated as the monthly household income divided by the square root of the number of persons in the household, categorized into quartiles by year and gender.

[2] Calculated as the weight in kilograms divided by the height in meters squared.

Supplementary Material 2. Weighted prevalence of impaired fasting glucose among Korean adults aged ≥30 years, 2007-2017.

	2007-2009			2010-2012			2013-2015			2016-2017			*p* for trend [2)]
	N	%	(SE)	n	**%**	(SE)	n	**%**	(SE)	**N**	%	(SE)	
Overall(years)													
≥30 (Unadjusted)	5525527	20.2	(0.5)	5724892	20.4	(0.5)	6908924	25.6	(0.5)	8453524	26.3	(0.6)	−
≥30 (Adjusted) [1)]	−	19.9	(0.5)	−	19.8	(0.5)	−	24.5	(0.5)	−	25.2	(0.6)	<0.001
30-39	1002555	13.1	(0.7)	891988	12.2	(0.8)	1126235	17.3	(1.0)	1228042	17.1	(1.0)	<0.001
40-49	1599552	20.6	(0.9)	1550971	20.2	(0.9)	1711604	24.2	(1.0)	2256811	27.5	(1.2)	<0.001
50-59	1399864	24.7	(1.0)	1596956	24.7	(0.9)	2142714	31.8	(1.0)	2297901	29.3	(1.1)	<0.001
60-69	883321	25.0	(1.0)	1026166	27.8	(1.0)	1179972	30.2	(1.1)	1545541	31.4	(1.2)	<0.001
≥70	640235	23.3	(1.1)	658812	22.8	(1.0)	748398	26.6	(1.2)	1125229	28.1	(1.3)	<0.001
Men(years)													
≥30 (Unadjusted)	3208532	23.8	(0.7)	3372956	24.5	(0.7)	4083283	30.9	(0.8)	5123122	32.1	(0.9)	−
≥30 (Adjusted) [1)]	−	23.7	(0.7)	−	24.0	(0.7)	−	30.0	(0.8)	−	31.3	(0.9)	<0.001
30-39	649603	16.5	(1.2)	609168	16.4	(1.3)	765859	23.2	(1.6)	847547	22.4	(1.5)	<0.001
40-49	1024141	25.9	(1.3)	1033719	26.3	(1.5)	1128813	32.0	(1.6)	1494469	36.0	(1.9)	<0.001
50-59	797261	27.9	(1.5)	930555	28.9	(1.5)	1237092	37.2	(1.6)	1452934	36.7	(1.8)	<0.001
60-69	448289	27.2	(1.5)	544071	31.0	(1.5)	599253	32.1	(1.5)	843311	35.1	(1.8)	0.001
≥70	289237	26.8	(1.8)	255443	22.3	(1.5)	352266	29.4	(1.8)	484861	29.4	(1.8)	0.052
Women(years)													
≥30 (Unadjusted)	2316995	16.7	(0.5)	2351936	16.5	(0.5)	2825642	20.5	(0.6)	3330402	20.5	(0.7)	−
≥30 (Adjusted) [1)]	−	16.2	(0.5)	−	15.5	(0.5)	−	19.0	(0.5)	−	19.0	(0.7)	<0.001
30-39	352952	9.5	(0.8)	282820	7.9	(0.8)	360377	11.3	(1.0)	380496	11.2	(1.1)	0.060
40-49	575411	15.1	(1.1)	517251	13.8	(1.0)	582791	16.4	(1.0)	762341	18.8	(1.3)	0.010
50-59	602603	21.5	(1.3)	666400	20.5	(1.1)	905623	26.5	(1.3)	844967	21.7	(1.3)	0.363
60-69	435032	23.0	(1.3)	482095	24.9	(1.5)	580719	28.5	(1.4)	702230	27.9	(1.5)	0.004
≥70	350997	21.1	(1.4)	403369	23.2	(1.3)	396133	24.6	(1.4)	640368	27.2	(1.8)	0.005

SE, standard error

[1)] Estimates are age-adjusted by direct standardization to the 2005 Korean census population.

[2)] Derived using weighted logistic regression by including the midpoint of each survey period as a continuous variable.

Supplementary Material 3. Crude weighted proportion of diabetes awareness, treatment, and control among Korean adults with diabetes aged ≥ 30 years, 2007-2017

	2007-2009		2010-2012		2013-2015		2016-2017	
	%	(SE)	%	(SE)	%	(SE)	%	(SE)
Awareness (diagnosed DM)								
Overall	72.6	(1.5)	72.7	(1.4)	70.5	(1.4)	72.3	(1.6)
Men	70.2	(2.1)	69.5	(2.1)	66.6	(2.1)	67.8	(2.1)
Women	75.5	(1.9)	76.7	(1.9)	75.5	(1.8)	77.8	(1.9)
Treatment (among prevalent DM)								
Overall	57.5	(1.5)	63.9	(1.5)	63.3	(1.5)	66.7	(1.6)
Men	53.6	(2.2)	60.8	(2.1)	59.1	(2.1)	62.4	(2.2)
Women	62.0	(2.1)	67.7	(2.1)	68.9	(1.8)	72.0	(2.1)
Control, A1C < 6.5% (among diagnosed DM)								
Overall	26.4	(1.6)	27.1	(1.6)	24.3	(1.4)	30.0	(1.7)
Men	28.1	(2.3)	25.5	(2.1)	24.2	(2.0)	28.7	(2.3)
Women	24.5	(1.9)	29.0	(2.4)	24.5	(2.1)	31.4	(2.3)
Control, A1C < 7% (among diagnosed DM)								
Overall	47.9	(1.8)	45.4	(1.8)	43.2	(1.7)	51.8	(1.8)
Men	49.0	(2.7)	44.5	(2.5)	43.8	(2.4)	50.4	(2.5)
Women	46.6	(2.5)	46.4	(2.4)	42.6	(2.5)	53.4	(2.5)
Control, Blood Pressure < 130/80 mmHg (among diagnosed DM)								
Overall	40.7	(1.8)	41.4	(1.6)	52.4	(1.7)	53.1	(1.9)
Men	38.8	(2.4)	41.1	(2.3)	49.4	(2.5)	51.1	(2.6)
Women	42.9	(2.3)	41.8	(2.4)	55.8	(2.3)	55.2	(2.6)
Control, Total Cholesterol < 200 mg/dL (among diagnosed DM)								
Overall	64.9	(1.8)	71.6	(1.5)	75.3	(1.5)	78.6	(1.4)
Men	71.9	(2.4)	75.3	(4.4)	77.7	(2.1)	80.7	(2.0)
Women	57.1	(2.4)	67.4	(2.4)	72.5	(2.2)	76.4	(2.1)

SE, standard error; DM, diabetes mellitus

Supplementary Material 4. Age-standardized weighted proportion of diabetes awareness, treatment, and control among Korean adults with diabetes aged ≥30 years, 2007-2017

	2007-2009		2010-2012		2013-2015		2016-2017		*p* for trend [2)]
	%	(SE)	%	(SE)	%	(SE)	%	(SE)	
Awareness (diagnosed DM) [1)]									
Overall	72.4	(1.5)	71.2	(1.4)	68.2	(1.5)	69.2	(1.6)	0.114
Men	71.7	(1.9)	69.3	(2.1)	66.2	(2.1)	66.4	(2.1)	0.041
Women	73.3	(2.1)	73.4	(2.2)	71.6	(2.2)	72.7	(2.3)	0.987
Treatment (among prevalent DM) [1)]									
Overall	57.2	(1.5)	62.2	(1.5)	60.8	(1.6)	63.5	(1.7)	0.008
Men	55.0	(2.1)	60.3	(2.0)	58.5	(2.1)	61.1	(2.2)	0.131
Women	59.7	(2.3)	64.6	(2.3)	64.7	(2.2)	66.3	(2.5)	0.014
Control, A1C < 6.5% (among diagnosed DM) [1)]									
Overall	25.6	(1.7)	25.8	(1.8)	23.5	(1.7)	28.0	(1.8)	0.314
Men	27.9	(2.3)	25.1	(2.4)	23.8	(2.2)	27.8	(2.5)	0.981
Women	22.8	(2.2)	26.1	(2.6)	23.9	(2.4)	28.9	(2.3)	0.109
Control, A1C < 7% (among diagnosed DM) [1)]									
Overall	46.6	(2.0)	44.4	(2.1)	40.4	(1.9)	48.9	(2.1)	0.234
Men	48.2	(2.6)	44.3	(2.8)	41.6	(2.5)	48.1	(2.8)	0.879
Women	45.5	(3.0)	42.8	(3.0)	40.1	(2.9)	50.6	(3.1)	0.108
Control, Blood Pressure < 130/80 mmHg (among diagnosed DM) [1)]									
Overall	41.1	(1.9)	43.0	(1.9)	50.9	(2.0)	53.2	(2.2)	<0.001
Men	38.6	(2.5)	41.8	(2.6)	46.2	(2.6)	49.5	(3.0)	<0.001
Women	45.4	(2.7)	46.8	(2.8)	59.6	(2.8)	60.6	(3.0)	<0.001
Control, Total Cholesterol < 200 mg/dl (among diagnosed DM) [1)]									
Overall	65.0	(1.8)	69.3	(1.9)	73.0	(1.9)	78.0	(1.7)	<0.001
Men	71.8	(2.4)	72.8	(2.4)	75.8	(2.3)	80.4	(2.1)	0.011
Women	59.3	(2.8)	64.6	(3.0)	69.4	(2.9)	74.8	(2.6)	<0.001

DM, diabetes mellitus; SE, standard error

[1)] All estimates are age-standardized to the subpopulation of persons who had diabetes in the KNHANES 2005.

[2)] Derived using weighted logistic regression by including the midpoint of each survey period as a continuous variable.
